# The impact of concomitant infective endocarditis in patients with spondylodiscitis and isolated spinal epidural empyema and the diagnostic accuracy of the modified duke criteria

**DOI:** 10.3389/fsurg.2023.1333764

**Published:** 2024-01-09

**Authors:** Mido Max Hijazi, Timo Siepmann, Ibrahim El-Battrawy, Assem Aweimer, Percy Schröttner, Martin Mirus, Dino Podlesek, Gabriele Schackert, Tareq A. Juratli, Ilker Y. Eyüpoglu, Andreas Filis

**Affiliations:** ^1^ Department of Neurosurgery, Division of Spine Surgery, Technische Universität Dresden, Faculty of Medicine, and University Hospital Carl Gustav Carus, Dresden, Germany; ^2^ Department of Neurology, Technische Universität Dresden, Faculty of Medicine, and University Hospital Carl Gustav Carus, Dresden, Germany; ^3^ Department of Cardiology and Angiology, Bergmannsheil University Hospitals, Ruhr University Bochum, Bochum, Germany; ^4^ Faculty of Medicine, and University Hospital Carl Gustav Carus, Institute for Microbiology and Virology, Technische Universität Dresden, Dresden, Germany; ^5^ Department of Anesthesiology and Intensive Care Medicine, Technische Universität Dresden, Faculty of Medicine, and University Hospital Carl Gustav Carus, Dresden, Germany

**Keywords:** spondylodiscitis, vertebral osteomyelitis, isolated spinal epidural empyema, infective endocarditis, modified duke criteria

## Abstract

**Background:**

The co-occurrence of infective endocarditis (IE) and primary spinal infections (PSI) like spondylodiscitis (SD) and isolated spinal epidural empyema (ISEE) has been reported in up to 30% of cases and represents a life-threatening infection that requires multidisciplinary management to be successful. Therefore, we aimed to characterize the clinical phenotypes of PSI patients with concomitant IE and furthermore to assess the accuracy of the modified Duke criteria in this specific population.

**Methods:**

We conducted a retrospective cohort study in consecutive SD and ISEE patients treated surgically at our University Spine Center between 2002 and 2022 who have undergone detailed phenotyping comprising demographic, clinical, imaging, laboratory, and microbiologic assessment. Comparisons were performed between PSI patients with IE (PSICIE) and without IE (PSIWIE) to identify essential differences.

**Results:**

Methicillin-susceptible *Staphylococcus aureu*s (MSSA) was the most common causative pathogen in PSICIE group (13 patients, 54.2%) and aortic valve IE was the most common type of IE (12 patients, 50%), followed by mitral valve IE (5 patients, 20.8%). Hepatic cirrhosis (*p* < 0.011; OR: 4.383; 95% CI: 1.405–13.671), septic embolism (*p* < 0.005; OR: 4.387; 95% CI: 1.555–12.380), and infection with *Streptococcus* spp. and *Enterococcus* spp. (*p* < 0.003; OR: 13.830; 95% CI: 2.454–77.929) were identified as significant independent risk factors for the co-occurrence of IE and PSI in our cohort. The modified Duke criteria demonstrated a sensitivity of 100% and a specificity of 66.7% for the detection of IE in PSI patients. Pathogens were detected more frequently via blood cultures in the PSICIE group than in the PSIWIE group (PSICIE: 23, 95.8% vs. PSIWIE: 88, 62.4%, *p* < 0.001). Hepatic cirrhosis (PSICIE: 10, 41.7% vs. PSIWIE: 33, 21.6%, *p* = 0.042), pleural abscess (PSICIE: 9, 37.5% vs. PSIWIE: 25, 16.3%, *p* = 0.024), sepsis (PSICIE: 20, 83.3% vs. PSIWIE: 67, 43.8%, *p* < 0.001), septic embolism (PSICIE: 16/23, 69.6% vs. PSIWIE: 37/134, 27. 6%, *p* < 0.001) and meningism (PSICIE: 8/23, 34.8% vs. PSIWIE: 21/152, 13.8%, *p* = 0.030) occurred more frequently in PSICIE than in PSIWIE patients. PSICIE patients received longer intravenous antibiotic therapy (PSICIE: 6 [4–7] w vs. PSIWIE: 4 [2.5–6] w, *p* < 0.001) and prolonged total antibiotic therapy overall (PSICIE: 11 [7.75–12] w vs. PSIWIE: 8 [6–12] w, *p* = 0.014). PSICIE patients spent more time in the hospital than PSIWIE (PSICIE: 43.5 [33.5–53.5] days vs. PSIWIE: 31 [22–44] days, *p* = 0.003).

**Conclusions:**

We report distinct clinical, radiological, and microbiological phenotypes in PSICIE and PSIWIE patients and further demonstrate the diagnostic accuracy of the modified Duke criteria in patients with PSI and concomitant IE. In the high-risk population of PSI patients, the modified Duke criteria might benefit from amending pleural abscess, meningism, and sepsis as minor criteria and hepatic cirrhosis as major criterion.

## 1 Introduction

Infective endocarditis (IE) refers to infection of a native or prosthetic heart valve, the endocardial surface, or an implanted cardiac instrument (1). Primary spinal infection (PSI) manifests mainly as spondylodiscitis (SD) or isolated spinal epidural empyema (ISEE), with SD resulting predominantly from hematogenous pathogen dissemination (2).

The co-occurrence of IE and SD has been reported up to 30% of cases (3–6), with endocarditis being more common in SD than in ISEE (2). Treatment of such life-threatening infections requires a multidisciplinary approach to achieve success.

In a patient with known IE and a new onset of back pain, imaging must be performed to rule out SD (6, 7). Similarly, any patient with SD should have a transthoracic echocardiogram (TTE) and if necessary, a transesophageal echocardiography (TEE) to exclude IE, especially if the patient has a proven gram-positive bacterium (GPB) or a heart valve replacement. The modified Duke criteria have been developed for all types of IE, although the accuracy in SD and ISEE patients has not yet been studied.

Behmanesh et al. demonstrated a tenfold higher rate of diagnosed IE after routine use of TEE in patients with known SD (3). TTE is recommended as first-line imaging in all patients with abnormal blood cultures, a new heart murmur, or suspected IE; due to sensitivity TEE should be performed in case of negative TTE but high suspicion of IE, equivocal TTE, prosthetic valves, cardiac devices, and positive TTE to detect other complications (8). Some authors suggest to perform TEE initially in patients with suspected IE who have an intermediate to high pretest probability of IE, including those with prosthetic heart valves, blood cultures growing *Staphylococci*, or an intracardiac device (9), although TTE may be equivalent and sometimes superior to TEE in the evaluation of the anterior cardiac regions, the cardiac apex, and the tricuspid valve (10–14).

Maintaining TTE as the first imaging examination should therefore always be considered depending on the clinical situation mentioned above, and TEE examination should then follow immediately regardless of the TTE findings due to the high prevalence of IE in SD (15). Cardiac computer tomography (CT) and 18F-FDG PET/CT represent additional options in the diagnostic algorithm (8, 15, 16).

The diagnosis of IE is based on the Duke criteria, described originally in 1994 and modified in 2000 (17, 18). Despite the widespread use of these criteria for the diagnosis of IE, there are significant limitations, and a substantial proportion of patients are classified as “possible IE” (19).

Previous studies have suggested that the time to diagnosis has a substantial impact on prognosis and mortality in PSI and IE and that the mortality rate is higher in patients with concomitant PSI and IE compared with PSI patients without IE (20–22). Baseline and risk factors, clinical course, causative pathogens, and surgical and anti-infective strategies for PSI patients with IE are poorly defined (7, 23).

Currently, there are several case reports of percutaneous mechanical debulking of vegetations or cysts in patients with IE or other cardiac infections using an AngioVac system (24, 25).

The accuracy of the modified Duke criteria in this subpopulation is unknown and treatment guidelines for this challenging subgroup of patients at risk are lacking to date. Therefore, we aimed to analyze the clinical and microbiological phenotype of patients with PSI and IE and we furthermore sought to identify risk factors for the co-occurrence of IE and PSI. Finally, we investigated the diagnostic accuracy of the modified Duke criteria in this specific subpopulation.

## 2 Materials and methods

### 2.1 Study design

We conducted a retrospective observational study to evaluate patients diagnosed with PSI with/without IE who underwent surgery at our University Neurosurgical Spine Centre between 2002 and 2022.

A total of 228 patients were identified. Fifty-one patients had to be excluded based on any of the following criteria:

- an echocardiographic assessment was not performed or documented (*n* = 31)
- only conservative treatment (*n* = 8)
- intradural infection (*n* = 12)

**Figure 1:**
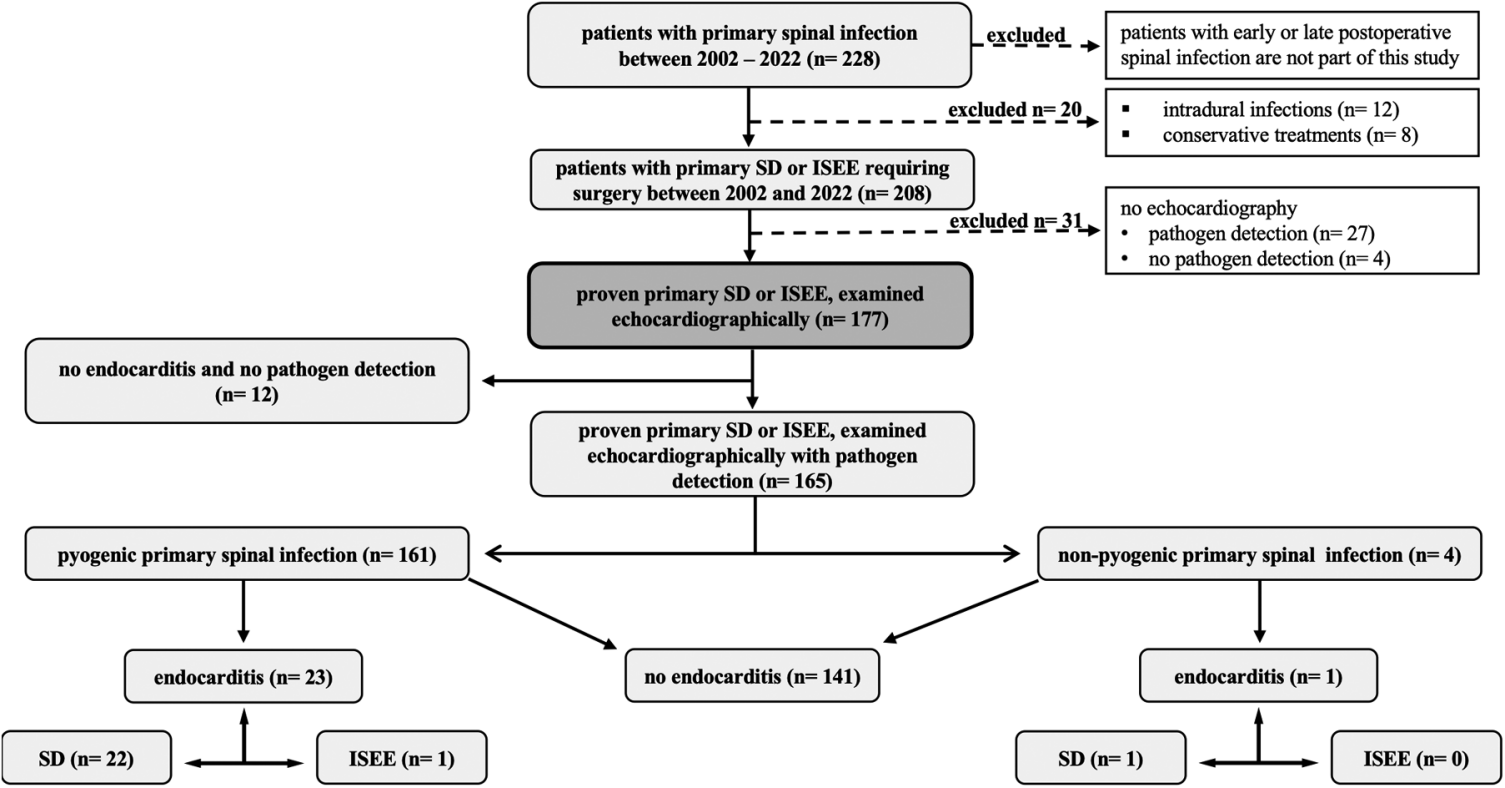
Study design. This figure shows our study design in 228 patients with primary spinal infection. Fifty-one patients were excluded due to intradural infection, only conservative treatment, or lack of echocardiography. SD, spondylodiscitis; ISEE, isolated spinal epidural empyema.

One hundred seventy-seven patients with primary SD and ISEE were included in the study. They underwent echocardiography, of whom 161 were diagnosed with pyogenic spinal infection and 4 with non-pyogenic infection, whereas no pathogen was detected in 12 patients (Figure 1).

### 2.2 Patient data

The study was conducted in accordance with the Helsinki Declaration. A positive vote of the responsible local ethics committee (Reference number BO-EK-17012022) has been obtained and an informed consent was waived. After case identification, patient data were extracted by reviewing electronic medical records using the ORBIS system (ORBIS, Dedalus, Bonn, Germany). Radiological data, including gadolinium contrast-enhancing magnetic resonance imaging (MRI), CT and/or x-ray were available for review in all cases.

Data collected included sex, age, type of PSI, causative pathogen, radiological findings on MRI and CT, and number of successful pathogen detections via blood cultures, intraoperative specimens, or CT-guided biopsies of the paravertebral psoas abscess. We also collected data on time passed to pathogen detection, type of antibiotic strategy, presence of psoas or pleural abscesses, spinal location of infection, time passed to surgery, use of intraoperative antibiotic irrigation, use of postoperative epidural suction-irrigation drainage, incidental dural tears, primary source of infection, surgical and antibiotic treatment, type of surgical procedure, risk factors (immunosuppression, diabetes mellitus, obesity, malignancy, liver cirrhosis, dialysis, stent or vascular prosthesis, artificial heart valve replacement, osteoporosis, rheumatoid arthritis or elevated rheumatoid factors, gout or elevated uric acid, chronic venous insufficiency, peripheral artery disease, and atrial fibrillation), disease-related complications (relapse rate, sepsis, septic embolism, meningism, success rate, reoperation due to surgical site infection, reoperation because of persistent empyema or spinal instability, disease-related mortality, hospitalization, and intensive care unit (ICU) stay), and classification according to the modified Duke criteria (definitive, possible, or rejected IE).

### 2.3 Clinical management

#### 2.3.1 Clinical, microbiological, and radiological diagnostics

The diagnosis of SD or ISEE was performed on the basis of medical history, clinical examination, fever, laboratory values (leukocyte count, C-reactive protein (CrP), and procalcitonin), typical radiological findings on MRI and CT, and pathogen detection on blood cultures, intraoperative specimens, or CT-guided biopsies of the paravertebral psoas abscess.

TTE was performed in all patients and the modified Duke criteria were applied, whereas in patients with GPB, possible or definite IE, TEE was performed according to the modified Duke criteria.

At least two blood cultures were obtained, and empirical or targeted antibiotic therapy (EAT, TAT) was performed. Tissue samples obtained during open surgery and samples taken by CT-guided biopsies were used for microbiological and histopathological analysis.

The diagnosis of ISEE or SD was determined in our multidisciplinary spine conference or neurosurgical-neuroradiological conference, and therapy was decided in collaboration with infectiologists, if possible. We have established a multidisciplinary spine board that includes neuroradiologists, neurosurgeons, trauma surgeons, orthopedic surgeons, and infectiologists, when appropriate, to determine the best treatment strategy for patients.

#### 2.3.2 Surgical and antibiotic management

Conservative treatment with intravenous antibiotics was usually the first-line of treatment, and surgical treatment was indicated in the absence of source control, epidural abscess, neurologic deficit, or spinal instability, with the type of surgical procedure determined in the multidisciplinary spine board or neurosurgical-neuroradiological conference.

ISEE patients underwent abscess evacuation with postoperative epidural suction-irrigation drainage or anterior cervical discectomy and fusion (ACDF) with abscess evacuation for abscesses ventral to the cervical spinal cord.

Patients with SD were treated with either abscess evacuation alone or one- or two-stage surgery. All patients with SD received a CT scan to assess bone structures and signs of instability. In case of biomechanical instability, patients underwent one-stage surgery for abscess evacuation and stabilization. Patients who initially had no instability and developed instability after abscess evacuation underwent two-stage surgery in terms of instrumentation. Surgical decision-making therefore depends on clinical experience and various defined radiological and clinical characteristics.

According to the clinical condition on admission and according to the recommendations of the local infectious diseases department, all patients were treated with either TAT or EAT strategy. However, it is important to note that most patients with EAT presented to us from peripheral hospitals and required immediate surgery; therefore, the number of EAT patients was high. EAT was changed to TAT after the causative pathogens were detected.

When surgical treatment was required in patients with proven concomitant endocarditis and PSI (SD or ISEE), abscess evacuation with placement of an epidural suction-irrigation drainage was performed in the first line if the patients had a stable cardiac state. In the case of spinal instability and a well-defined indication for instrumentation, we performed a single-stage surgery. In case of cardiac decompensation, cardiologic and cardiosurgical evaluation was performed first, with medical treatment and surgical sanitation of endocarditis, if necessary, followed by re-evaluation of possible spinal surgery.

Intravenous antibiotic treatment was administered to ISEE patients for approximately 2 weeks and additional oral antibiotic treatment for 2 to 4 weeks, whereas SD patients received intravenous antibiotic therapy for approximately 4 to 6 weeks and additional oral antibiotic therapy for 6 to 8 weeks. Clinical and radiological follow-up was performed in all patients who complied with our recommendation at 3, 6, and 12 months after hospital discharge.

### 2.4 Case illustration of infective endocarditis

**Figure 2:**
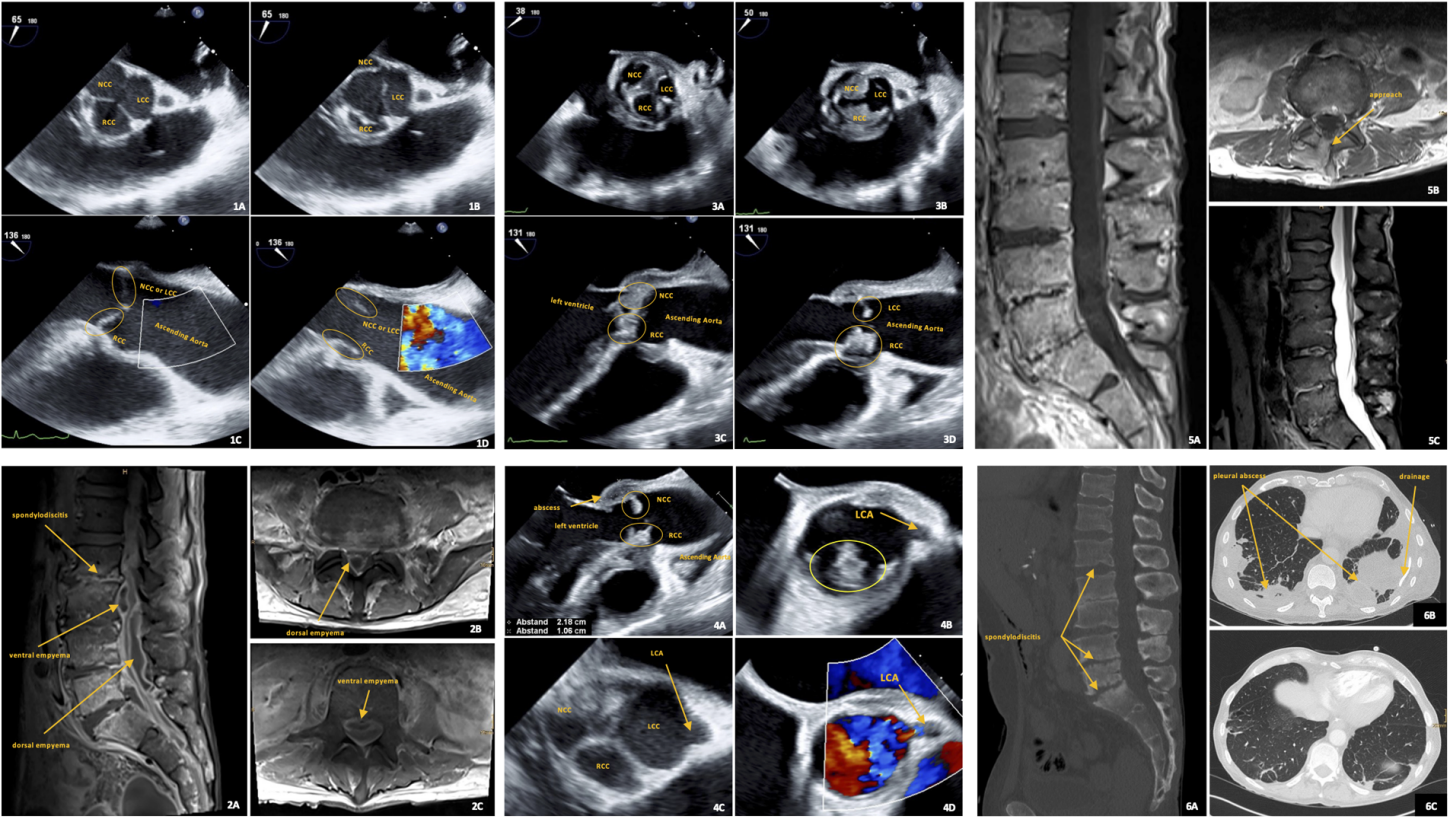
Case presentation. Case presentation of a patient with concurrent spondylodiscitis and endocarditis. TEE images of the midesophageal aortic valve short axis: during diastole (**1A**: aortic valve closed) and systole (**1B**: aortic valve open). TEE images of the midesophageal aortic valve long axis: during diastole (**1C**) and systole (**1D**). Images **1A**–**1D** show the patient's aortic valve without evidence of endocarditis at the time when spondylodiscitis with ventral and dorsal empyema was clinically, radiologically, and histopathologically confirmed as shown by the sagittal (image: **2A**) and axial (images: **2B**, **2C**) fat-saturated contrast-enhanced T1-weighted MR images. TEE images **3A**, **3B**, **3C**, and 3D show severe aortic valve endocarditis six months later, the three aortic valve cusps in the closed valve image 3A with thickened commissures between them compared to image **1A** and the thickened NCC and RCC cusps with adherent masses in the open valve image **3B** indicate vegetations, LCC seems to be less affected. Images **3C** and **3D** also show massive protrusion of vegetations in the left ventricle compared with images **1C** and **1D**. TEE image **4A** shows the extent of the abscess in the region of the aortomitral junction. TEE images **4B**, **4C** and **4D** show left coronary artery (LCA) outflow near the aortic valve. TEE B-mode image (**4C**) and duplex image (**4D**) of the midesophageal aortic valve short axis show the LCA in the patient without endocarditis. Image 4B shows the outflow of the LCA in this patient, which remains open despite manifest endocarditis in the region of the RCC six months later. The spatial proximity of aortic valve endocarditis to LCA outflow shown here highlights the risk of obstruction of the LCA by large vegetations. This may lead to acute myocardial ischemia with appropriate consequences. Sagittal (**5A**) and axial (**5B**) fat-saturated contrast-enhanced T1-weighted and sagittal T2-weighted (**5C**) MR images show the lumbar spine as it progresses after 5 months of local and systematic antibiotic treatment and surgical management of spondylodiscitis with empyema. The sagittal reformatted CT image (**6A**) shows the extent of bone destruction in the lumbar spine and the onset of bone consolidation. CT images (**6B** and **6C**) show the multiple lung abscesses before drainage placement and after systemic and local treatment. Cusp of the aortic valve: NCC, non-coronary cusp; LCC, left coronary cusp; RCC, right coronary cusp.

We present a 66-year-old male patient with infective endocarditis of the aortic valve who had an abscess at the aortic root and endocarditis of the mitral valve with perforation of the posterior mitral leaflet (PML). The patient has known gout and underwent pancreatectomy, splenectomy, subtotal gastrectomy, and subtotal colectomy due to a neuroendocrine tumor in the left upper abdomen with liver metastases. He had a port implanted in the left subclavian vein, but 2 months later he developed sepsis due to a port infection caused by *Staphylococcus epidermidis*. The patient had paraparesis, and MRI showed extensive spondylodiscitis with an epidural abscess ventrodorsally at the level of L2 to S2. *Staphylococcus epidermidis* was both times detected via blood culture and intraoperatively when an epidural abscess was evacuated, an infected port was removed, and a pleural drainage was inserted for several pleural abscesses. The abscess was drained via an interlaminar approach at the level of L3/4 on the right, and an epidural suction-irrigation drainage was inserted. The patient was treated locally and systematically with antibiotics and could be transferred for rehabilitation. He was first treated with vancomycin intravenously for 3 weeks and then with linezolid and daptomycin intravenously for 5 weeks because of impaired renal function before being discharged home. Follow-up after completion of antibiotic treatment was unremarkable (TTE, MRI of spine and CT of thorax and abdomen, infectious parameters). After about 6 months, the patient's general condition deteriorated and he developed dyspnea, cough, fever, palpitations, and weight loss. He was admitted to the emergency room, where severe sepsis was again evident. MRI of the spine performed showed no relevant new abscess or discitis, but TEE showed a new-onset infective double-valve endocarditis. Blood cultures again showed multiple *Staphylococcus epidermidis* and *Staphylococcus caprae*. However, the CT of the thorax showed no abscesses in the lungs. Treatment with daptomycin was resumed, and MRI examination of the neurocranium due to seizures showed multiple septic emboli. From a cardiac surgical point, the indication for surgical repair with valve replacement was given for aortic and mitral insufficiency III°. Aortic and mitral valve replacement was performed by xenotransplantation of 21 and 27 mm SJM Epic (Supra) and stabilization of the aortic annulus by a tubular prosthetic ring (Calamari) and then the patient was discharged to home. Antibiotic therapy with daptomycin was continued for a further 4 weeks. A new port was implanted and a urinary tract infection and pneumonia occurred in the course of the treatment, which was followed by renewed antibiotic therapy with Piperacillin/tazobactam. Another port explantation was performed and a shaldon catheter was inserted for acute renal failure. A hematoma formed during bleeding from the superficial femoral artery, subsequently the hematoma was surgically evacuated. The antibiotics were stopped after 2 weeks. The acute renal failure regressed and the patient could be mobilized to the ward floor. The TEE check revealed paraprosthetic aortic valve insufficiency. Cardiac surgery was therefore recommended. The patient and his relatives decided against surgery and renewed antibiotic treatment. The patient was discharged home with a palliative concept and died a few weeks later (Figure 2).

### 2.5 Statistical analysis

The SPSS software package (SPSS Statistics 28, IBM, Armonk, New York, USA) was used for all statistical analyses. Descriptive statistics were used, and categorical variables were compared between PSICIE and PSIWIE using Fisher's exact tests or chi-square tests. Numeric variables were compared with Mann-Whitney U tests. All statistical tests were two-sided tests, and a *p*-value of *p* < 0.05 was considered statistically significant. Univariate and multivariate analyses were conducted to identify independent risk factors. Sensitivity = (true positives) / (true positives + false negatives) and specificity = (true negatives) / (true Negatives + false positives) (26).

## 3 Results

### 3.1 Demographic data and patient characteristics

We included 177 patients (men: 116, 65.5%) with 90 patients over 65 years (50.8%), of whom 125 presented with SD (70.6%) and 52 with ISEE (29.4%) (Table 1). PSI was identified in 161 patients (91.0%) and non-pyogenic spinal infection in 4 patients (2.2%), whereas the causative pathogen could not be detected in 12 patients (6.8%). Methicillin-susceptible *Staphylococcus aureus* (MSSA) was isolated in 53.4% (86/161), *Streptococcus* spp. and *Enterococcus* spp. in 18.0% (29/161), Enterobacterales in 11.2% (18/161), and Coagulase-negative *Staphylococci* (CoNS) in 9.9% (16/161). GPB represented 86.3% of cases (139/161) and Gram-negative bacteria (GNB) 13.7% of cases (22/161).

**Table 1:** Patient characteristics.

Characteristics	*N* = 177	Percentage
Baseline factors		
Males	116	65.5%
Females	61	34.5%
Age > 65	90	50.8%
Spondylodiscitis	125	70.6%
Isolated spinal epidural empyema	52	29.4%
Pathogen		
Pyogenic spinal infection	161	91.0%
Non-pyogenic spinal infection	4	2.2%
Known causative pathogens	165	93.2%
Unknown causative pathogens	12	6.8%
MSSA	86/161	53.4%
*Streptococcus* spp. & *Enterococcus* spp.	29/161	18.0%
Enterobacterales	18/161	11.2%
CoNS	16/161	9.9%
Gram-positive bacteria	139/161	86.3%
Gram-negative bacteria	22/161	13.7%
Diagnostic and therapy		
Surgery	177	100%
Blood cultures	177	100%
CT-guided biopsy of psoas abscess	59/110	53.6%
Detected via blood cultures	111/165	67.3%
Detected via intraoperative specimens	137/165	83.0%
Detected via CT-guided biopsy of psoas abscess	35/59	59.3%
EAT	116	65.5%
TAT	61	34.5%
Known primary infectious sources	127	71.8%
Paravertebral psoas abscess	110	62.1%
Pleural abscess	34	19.2%
CS	48	27.1%
TS	70	39.5%
LS	120	67.8%
Multiple localizations of spinal parts	49	27.7%
Time to surgery	1 [1–4] d	–
Time to pathogen detection	4 [3–6] d	–
Incidental dural tears	23	13.0%
Intraoperative antibiotic Irrigation	148	83.6%
Suction-irrigation drainage	121	68.4%
Only abscess evacuation	75	42.4%
One-staged surgery (evacuation and concomitant fixation)	68	38.4%
Two-staged surgery (evacuation followed by fixation)	34	19.2%
Intravenous antibiotic duration	4 [3–6] w	–
Total antibiotic duration	8 [6–12] w	–
Risk factors		
Immunosuppression	31	17.5%
Diabetes mellitus	66	37.3%
Obesity (BMI > 30 kg/m^2^)	54	30.5%
Malignancy	34	19.2%
Hepatic cirrhosis	43	24.3%
Dialysis	7	4%
Stent or vascular prosthesis	15	8.5%
Artificial heart valve replacement	12	6.8%
Osteoporosis	77/164	47.0%
Rheumatoid arthritis or increased rheumatic factors	34/87	39.1%
Gout or increased uric acid	42/87	48.3%
Chronic venous insufficiency	5	2.8%
Peripheral artery disease	10	5.6%
Atrial fibrillation	42	23.7%
Disease-related complications		
Disease-related mortality	9	5.1%
Relapse rate	26/116	22.4%
Sepsis	87	49.2%
Septic embolism elsewhere	53/157	33.8%
Meningism	29/175	16.6%
Infective endocarditis with vegetation in echocardiography	24	13.6%
Reoperation due surgical site infections	26	14.7%
Reoperation due to empyema persistence or instability	47	26.6%
Hospital stay	33 [19–44] d	–
ICU stay	4 [0–16] d	–
Modified Duke-criteria		
Definitive IE based on modified Duke-criteria	32	18.1%
Possible IE based on modified Duke-criteria	43	24.3%
Rejected IE based on modified Duke-criteria	102	57.6%

SD, spondylodiscitis;ISEE, isolated spinal epidural empyema; MSSA, methicillin-susceptible *Staphylococcus aureu*s; CoNS, coagulase-negative *Staphylococci*; CT, computer tomography; EAT, empirical antibiotic therapy; TAT, targeted antibiotic therapy; CS, cervical spine; TS, thoracic spine; LS, lumbar spine; BMI, body mass index; ICU, intensive care unit; IE, infective endocarditis; d, days; w, weeks. Bold values are significant results *(p* < 0.05) as indicated in the methods.

Multiple blood cultures and intraoperative samples were taken from all patients, whereas 59 tappable patients (53.6%) out of 110 patients with paravertebral psoas abscess (62.1%) underwent CT-guided biopsy. Pathogens were detected in 67.3% via blood cultures (111/165), in 83.0% via intraoperative specimens (137/165), and in 59.3% via CT-guided biopsy of a paravertebral psoas abscess (35/59).

EAT was initially performed in 116 patients (65.5%) and later changed to TAT as soon as the underlying bacterial species was known, while 61 patients (34.5%) were treated directly with a TAT. The primary infectious sources were identified in 127 of the cases (71.8%). Pleural abscess occurred in 34 patients (19.2%).

The infection was located in the cervical spine in 48 patients (27.1%), in the thoracic spine in 70 patients (39.5%), and in the lumbar spine in 120 patients (67.8%), while 49 of these cases had multiple locations in the spinal parts (27.7%).

The time from diagnosis of infection on MRI to performing surgery was 1 [1–4] days (median [interquartile), whereas the time to pathogen detection was 4 [3–6] days. Incidental dural tears occurred in 23 patients (13.0%), 148 patients (83.6%) were irrigated intraoperatively with local antibiotic (gentamicin, vancomycin, or both), whereas 121 patients were treated with postoperatively epidural suction-irrigation drainage 121 (68.4%).

Abscess evacuation alone was performed in 75 patients (42.4%), whereas single-stage surgery (evacuation and concomitant fixation, 38.4%) was performed in 68 patients and two-stage surgery (evacuation followed by fixation, 19.2%) was achieved in 34 patients. Patients received intravenous antibiotics for approximately 4 [3–6] weeks and a total antibiotic duration of approximately 8 [6–12] weeks.

Among the co-morbidities, 31 patients had immunosuppression (17.5%), 66 patients had a medical history of diabetes mellitus (37.3%), 54 patients were overweight with body mass index (BMI) over 30 kg/m^2^ (30.5%), 34 patients had malignancy (19.2%), 43 patients suffered from liver cirrhosis (24.3%), 7 patients required dialysis (4.0%), 15 patients had a stent or vascular prosthesis (8.5%), 12 patients had an artificial heart valve replacement (6.8%), 77 of 164 patients were osteoporotic (47.0%), 34 of 87 patients suffered from rheumatoid arthritis or had elevated rheumatoid factors (39.1%), 42 of 87 patients had gout or elevated uric acid (48.3%), 5 patients showed chronic venous insufficiency (2.8%), 10 patients had peripheral artery disease (5.6%), and 42 patients had atrial fibrillation (23.7%).

A total of nine patients (5.1%) died from the diseases and their complications, and 26 of 116 patients (22.4%) relapsed. Sepsis was observed in 87 patients (49.2%), septic embolism in 53 of 157 patients (33.8%), meningism in 29 patients (16.6%), and infective endocarditis with vegetations on echocardiography in 24 patients (13.6%). Reoperation due to surgical site infection was performed in 26 patients (14.7%), and reoperation due to persistent empyema and spinal instability was reported in 47 patients (26.6%). Median hospital stay was 33 [19–44] days and length of stay in the intensive care unit (ICU) was 4 [0–16] days.

Using the modified Duke criteria, 32 patients (18.1%) were considered as definitive IE, 43 patients (24.3%) as possible IE, and 102 as rejected IE (57.6%).

### 3.2 PSICIE vs. PSIWIE

There were significantly more SD patients in the PSICIE group (*n* = 23, 95.8%) than ISEE patients (*n* = 1, 4.2%, *p* = 0.003). Gender, age, and microbial groups such as MSSA, Enterobacterales, and CoNS demonstrated no difference between the two groups. In contrary, the *Streptococcu*s spp. & *Enterococcus* spp. subgroup occurred significantly more often in PSICIE (n = 8, 34.8%) than PSIWIE (n = 21, 15.2%, *p* = 0.037). The PSICIE group consisted only of GPB compared to the PSIWIE group (PSICIE: 23, 100.0% vs. PSIWIE: 116, 84.1%, *p* = 0.046) (Table 2).

**Table 2:** PSICIE vs. PSIWIE.

Characteristics	PSICIE	PSIWIE	*p*-value
Baseline factors			
Males	19 (79.2%)	97 (63.4%)	0.168^a^
Females	5 (20.8%)	56 (36.6%)	
Age > 65	13 (54.2%)	77 (50.3%)	0.827^a^
Spondylodiscitis	23 (95.8%)	102 (66.7%)	0.003^a^
Isolated spinal epidural empyema	1 (4.2%)	51 (33.3%)	
Pathogen			
Pyogenic spinal infection	23 (95.8%)	150 (98.0%)	0.445^a^
Non-pyogenic spinal infection	1 (4.2%)	3 (2.0%)	
MSSA	13 (56.5%)	73 (52.9%)	0.824^a^
*Streptococcus* spp. & *Enterococcus* spp.	8 (34.8%)	21 (15.2%)	0.037^a^
Enterobacterales	0 (0.0%)	18 (13.0%)	0.078^a^
CoNS	2 (8.7%)	14 (10.1%)	1.000^a^
Gram-positive bacteria	23 (100.0%)	116 (84.1%)	0.046^a^
Gram-negative bacteria	0 (0.0%)	22 (15.9%)	
Diagnostic and therapy			
Detected via blood cultures	23 (95.8%)	88 (62.4%)	<0.001^a^
Detected via intraoperative specimens	18 (75%)	119 (84.4%)	0.251^a^
Detected via CT-guided biopsy of psoas abscess	6/8 (75.0%)	29/51 (56.9%)	0.453^a^
EAT	8 (33.3%)	108 (70.6%)	<0.001^a^
TAT	16 (66.7%)	45 (29.4%)	
Polymicrobial infections	1 (4.2%)	7 (5.0%)	1.000^a^
Paravertebral psoas abscess	16 (66.7%)	94 (61.4%)	0.659^a^
CT-guided biopsy of psoas abscess	8 (33.3%)	54 (35.3%)	1.000^a^
Pleural abscess	9 (37.5%)	25 (16.3%)	0.024^a^
CS	7 (29.2%)	41 (26.8%)	0.808^a^
TS	10 (41.7%)	60 (39.2%)	0.826^a^
LS	19 (79.2%)	101 (66.0%)	0.245^a^
Multiple localizations of spinal parts	10 (41.7%)	39 (25.5%)	0.139^a^
Time to surgery	2 [1–4] d	2 [1–7] d	0.624^b^
Time to pathogen detection	3.5 [2–5] d	5 [3–8.5] d	0.054^b^
Incidental dural tears	6 (25.0%)	17 (11.1%)	0.067^a^
Intraoperative antibiotic Irrigation	19 (79.2%)	129 (84.3%)	0.554^a^
Suction-irrigation drainage	17 (70.8%)	104 (68.0%)	1.000^a^
Only abscess evacuation	9 (37.5%)	66 (43.1%)	0.662^a^
One-staged surgery (evacuation and concomitant fixation)	11 (45.8%)	57 (37.3%)	0.500^a^
Two-staged surgery (evacuation followed by fixation)	4 (16.7%)	30 (19.6%)	1.000^a^
Intravenous antibiotic duration	6 [4–7] w	4 [2.5–6] w	<0.001^b^
Total antibiotic duration	11 [7.75–12] w	8 [6–12] w	0.014^b^
Primary infectious sources			
Skin infection	6 (30.0%)	25 (23.4%)	0.231^a^
Foreign body associated infection	7 (35.0%)	16 (15.0%)	
Epidural administration	2 (10.0%)	19 (17.8%)	
Respiratory tract	3 (15.0%)	17 (15.9%)	
Urinary tract	0 (0.0%)	11 (10.3%)	
Gastrointestinal tract	1 (5.0%)	8 (7.5%)	
Retropharyngeal and prevertebral infection	0 (0.0%)	6 (5.6%)	
Odontogenic	0 (0.0%)	4 (3.7%)	
Immunosuppression	1 (5.0%)	1 (0.9%)	
Risk factors			
Immunosuppression	5 (20.8%)	26 (17.0%)	0.577^a^
Diabetes mellitus	9 (37.5%)	57 (37.3%)	1.000^a^
Obesity (BMI > 30 kg/m^2^)	5 (20.8%)	49 (32.0%)	0.344^a^
Malignancy	5 (20.8%)	29 (19.0%)	0.785^a^
Hepatic cirrhosis	10 (41.7%)	33 (21.6%)	0.042^a^
Dialysis	2 (8.3%)	5 (3.3%)	0.242^a^
Stent or vascular prosthesis	1 (4.2%)	14 (9.2%)	0.697^a^
Artificial heart valve replacement	3 (12.5%)	9 (5.9%)	0.211^a^
Osteoporosis or low density in CT (HU < 100)	8/22 (36.4%)	69/142 (48.6%)	0.361^a^
Rheumatoid arthritis or increased rheumatic factors	8/16 (50.0%)	26/71 (36.6%)	0.398^a^
Gout or increased uric acid	6/13 (46.2%)	36/74 (48.6%)	1.000^a^
Chronic venous insufficiency	0 (0.0%)	5 (3.3%)	1.000^a^
Peripheral artery disease	3 (12.5%)	7 (4.6%)	0.138^a^
Atrial fibrillation	7 (29.2%)	35 (22.9%)	0.606^a^
Disease-related complications			
Disease-related mortality	3/24 (12.5%)	6/153 (3.9%)	0.106^a^
Relapse rate	5/12 (41.7%)	21/104 (20.2%)	0.137^a^
Sepsis	20 (83.3%)	67 (43.8%)	<0.001^a^
Septic embolism elsewhere	16/23 (69.6%)	37/134 (27.6%)	<0.001^a^
Meningism	8/23 (34.8%)	21/152 (13.8%)	0.030 (1)
Success rate	10 (41.7%)	85 (55.6%)	0.271^a^
Reoperation due surgical site infections	4 (16.7%)	22 (14.4%)	0.759^a^
Reoperation due to empyema persistence or instability	7 (29.2%)	40 (26.1%)	0.805^a^
Hospital stay	43.5 [33.5–53.5] d	31 [22–44] d	0.003^b^
ICU stay	2.5 [0–8] d	1 [0–8] d	0.614^b^
Modified Duke-Criteria			
Definitive IE based on modified Duke-Criteria	24 (100.0%)	8 (5.2%)	< 0.001^a^
Possible IE based on modified Duke-Criteria	0 (0.0%)	43 (28.1%)	
Rejected IE based on modified Duke-Criteria	0 (0.0%)	102 (66.7%)	

PSICIE, primary spinal infection concomitant infective endocarditis; PSIWIE, primary spinal infection without infective endocarditis; MSSA, methicillin-susceptible *Staphylococcus aureu*s; CoNS, coagulase-negative *Staphylococci*; CT, computer tomography; EAT, empirical antibiotic therapy; TAT, targeted antibiotic therapy; CS, cervical spine; TS, thoracic spine; LS, lumbar spine; BMI, body mass index; HU, Hounsfield unit; ICU, intensive care unit; IE, infective endocarditis.

^a^ Fisher's exact test.

^b^ Mann–Whitney *U* test. Bold values are significant results (*p *< 0.05) as indicated in the methods.

Pathogens were detected more frequently via blood cultures in the PSICIE group (n = 23, 95.8%) than in the PSIWIE group (n = 88, 62.4%, *p* < 0.001). However, detection via intraoperative specimens (PSICIE: 18, 75% vs. PSIWIE: 119, 84.4%, *p* = 0.251) and via CT-guided biopsy of the psoas abscess (PSICIE: 6/8, 75.0% vs. PSIWIE: 29/51, 56.9%, *p* = 0.453) showed no difference between the two groups. PSICIE patients were treated more frequently with TAT strategy compared to PSIWIE patients (PSICIE: 16, 66.7% vs. PSIWIE: 45, 29.4%, *p* < 0.001), the opposite was observed for EAT strategy (PSICIE: 8, 33.3% vs. PSIWIE: 108, 70.6%, *p* < 0.001).

Polymicrobial infections, paravertebral psoas abscess, and CT-guided biopsy of paravertebral psoas abscess were equally distributed in both groups, however, PSICIE patients had more pleural abscesses than PSIWIE (PSICIE: 9, 37.5% vs. PSIWIE: 25, 16.3%, *p* = 0.024).The localization of infection in the spine, time to surgery, time to pathogen detection, the occurrence of incidental dural tears during surgery, the application of intraoperative antibiotic irrigation, the use of postoperative epidural suction-irrigation drainage, and the type of surgical procedure showed no significant difference between the two groups.

The PSICIE group had longer intravenous antibiotic duration (PSICIE: 6 [4–7] w vs. PSIWIE: 4 [2.5–6] w, *p* < 0.001) and longer total antibiotic duration (PSICIE: 11 [7.75–12] w vs. PSIWIE: 8 [6–12] w, *p* = 0.014). There was no between-group difference in the primary sources of infection (*p* = 0.231).

There was no significant difference in the distribution of primary sources of infection between the PSICIE and PSIWIE groups.

Hepatic cirrhosis was the only risk factor found more frequently in PSICIE patients than PSIWIE (PSICIE: 10, 41.7% vs. PSIWIE: 33, 21.6%, *p *= 0. 042), whereas the other risk factors such as immunosuppression, diabetes mellitus, obesity (BMI > 30 kg/m^2^), malignancy, dialysis, stent or vascular prosthesis, artificial heart valve replacement, osteoporosis, rheumatoid arthritis or elevated rheumatoid factors, gout or elevated uric acid, chronic venous insufficiency, peripheral arterial disease, and atrial fibrillation showed no difference between the groups.

Disease-related complications such as sepsis (PSICIE: 20, 83.3% vs. PSIWIE: 67, 43.8%, *p* < 0.001), septic embolism (PSICIE: 16/23 (69.6%) vs. PSIWIE: 37/134, 27. 6%, *p* < 0.001), and meningism (PSICIE: 8/23, 34.8% vs. PSIWIE: 21/152, 13.8%, *p* = 0.030) occurred more frequently in PSICIE patients than in PSIWIE patients. There was no difference between the two groups in disease-related mortality, relapse rate, success rate, reoperation due to surgical site infection, reoperation due to persistence of empyema or spinal instability.

PSICIE patients spent longer time in hospital (PSICIE: 43.5 [33.5–53.5] d vs. PSIWIE: 31 [22–44] d, *p* = 0.003), while ICU stay showed no significant difference between the two groups (PSICIE: 2.5 [0–8] d vs. PSIWIE: 1 [0–8] d, *p* = 0.614).

The modified Duke criteria showed a significant difference between the two groups as follows: definitive IE (PSICIE: 24, 100.0% vs. PSIWIE: 8, 5.2%), possible IE (PSICIE: 0, 0.0% vs. PSIWIE: 43, 28.1%), and rejected IE (0, 0.0% vs. PSIWIE: 102, 66.7%, *p* < 0.001).

### 3.3 Accuracy of modified duke-criteria in PSI patients

The modified Duke criteria have a sensitivity of 100% and a specificity of 66.7% for the diagnosis of IE in PSI patients.

$$\begin{array}{c} \mathrm{Sensitivity}\;\mathrm{value}=(24)/(24+0)=1\times 100\text{\%}=100\text{\%}\;\\ \mathrm{Specificity}\;\mathrm{value}=(102)/(102+51)=0.667\times 100\text{\%}=66.7\text{\%}\; \end{array}$$

#### 3.3.1 Causative pathogens and type of infective endocarditis

##### 3.3.1.1 Causative pathogens

**Figure 3:**
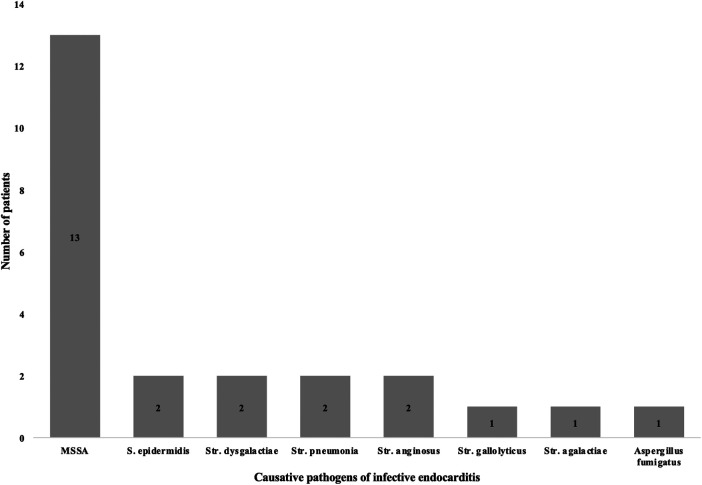
Causative pathogens in infective endocarditis. This figure represents the causative pathogens in infective endocarditis in PSI patients. MSSA: methicillin-susceptible *Staphylococcus aureu*s, S.: *Staphylococcus*, Str.: *Streptococcus*.

MSSA was the most common causative pathogen in PSICIE patients (*n* = 13, 54.2%), followed by *Staphylococcus epidermidis* (*n* = 2, 8.3%), *Streptococcus dysgalactiae* (*n* = 2, 8.3%), *S. pneumonia* (*n* = 2, 8.3%), *S. anginosus* (*n* = 2, 8.3%), *S. gallolyticus* (*n* = 1, 4.2%), *S. agalactiae* (*n* = 1, 4.2%), and *Aspergillus fumigatus* (*n* = 1, 4.2%) (Figure 3).

##### 3.3.1.2 Endocarditis type

**Figure 4:**
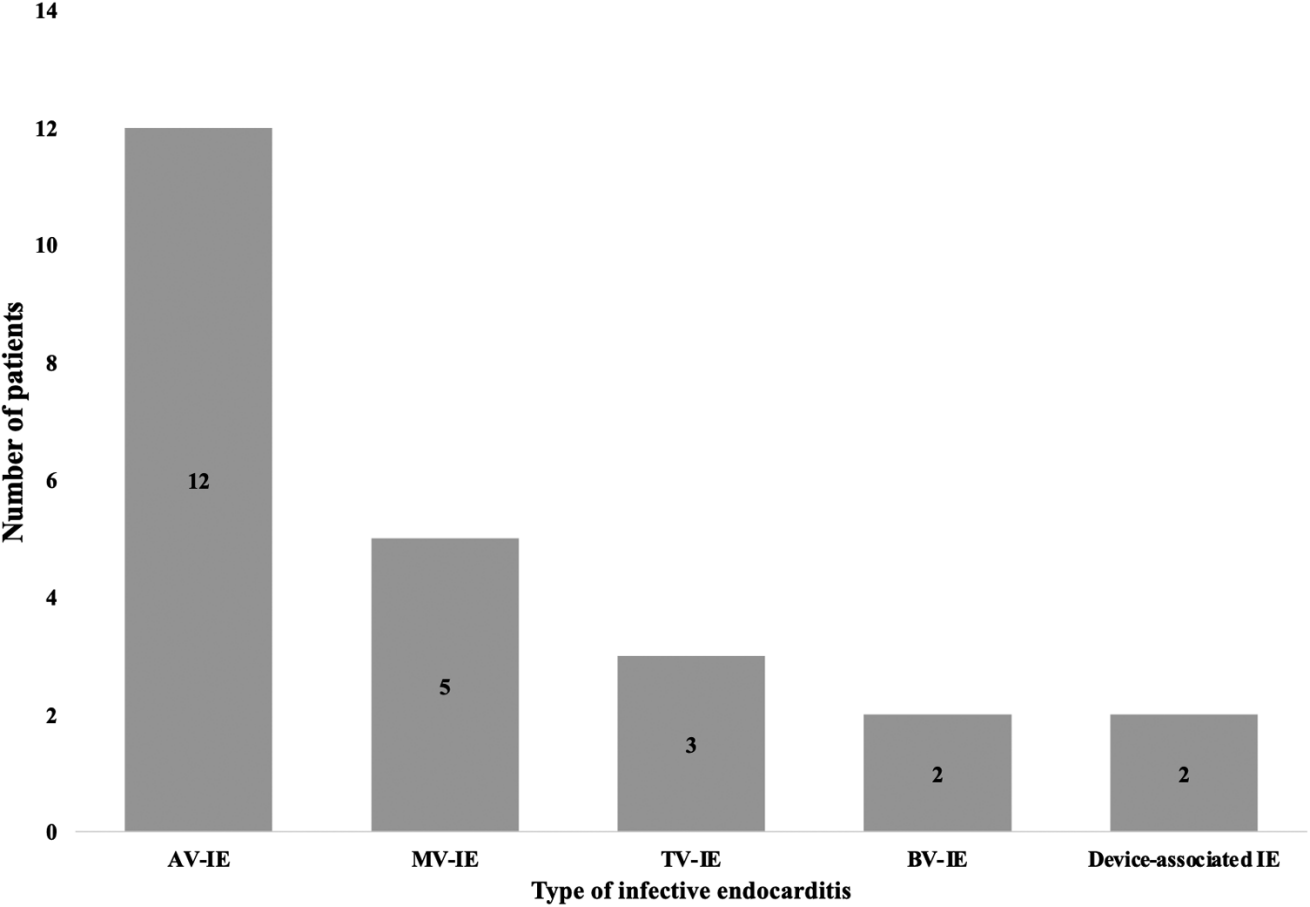
Type of infective endocarditis. This figure illustrates the type of infective endocarditis (IE) with reference to the affected heart valve. AV, Aortic valve; MV, mitral valve; TV, tricuspid valve; BV, double valve/ bivalve.

The aortic valve IE was the most common type of IE in PSICIE patients (*n* = 12, 50%), followed by mitral valve IE (*n* = 5, 20.8%), tricuspid valve IE (*n* = 3, 12.5%), double valve IE (*n* = 2, 8.3%), and device-associated IE (*n* = 3, 12.5%) (Figure 4).

### 3.4 Multivariate regression analysis for the development of IE in PSI patients

Multivariate binary logistic regression analyses are summarized in (Table 3). Infection with *Streptococcus* spp. and *Enterococcus* spp. (*p* < 0.003; OR: 13.830; 95% CI: 2. 454–77.929), septic embolism (*p* < 0.005; OR: 4.387; 95% CI: 1.555–12.380), and hepatic cirrhosis (*p* < 0.011; OR: 4.383; 95% CI: 1.405–13.671) were identified as a significant independent risk factors for co-occurrence of IE and PSI in our cohort.

**Table 3:** Multivariate analysis to identify independent risk factors for development of infective endocarditis.

Variables	Multivariate logistic regression	
	OR (95% CI)	*p*-value
Age	0.993 (0.932–1.058)	0.822
Males	2.321 (0.697–7.730)	0.170
SD	4.327 (0.505–37.074)	0.181
CS	0.857 (0.167–4.393)	0.854
TS	0.925 (0.072–11.962)	0.983
LS	1.926 (0.546–6.787)	0.308
More than one part of the spine	1.416 (0.360–5.560)	0.618
Methicillin-sensitive *Staphylococcus aureus*	3.703 (0.852–16.091)	0.081
*Streptococcus* spp. and *Enterococcus* spp.	13.830 (2.454–77.929)	**0**.**003**
Paravertebral psoas abscess	0.509 (0.121–2.143)	0.357
Pleural abscess	2.627 (0.850–8.120)	0.093
Sepsis	1.998 (0.432–9.239)	0.375
Septic embolism	4.387 (1.555–12.380)	**0**.**005**
Meningism	1.653 (0.416–6.570)	0.475
Obesity (BMI > 30 kg/m^2^)	1.121 (0.253–4.967)	0.880
Immunosuppression	1.499 (0.339–6.623)	0.593
Diabetes mellitus	1.307 (0.381–4.482)	0.670
Malignancy	0.469 (0.069–3.187)	0.439
Hepatic cirrhosis	4.383 (1.405–13.671)	**0**.**011**
Dialysis	0.339 (0.028–4.161)	0.398
Stent or vascular prosthesis	0.487 (0.46–5.180)	0.551
Artificial heart valve replacement	1.191 (0.136–10.406)	0.874
Peripheral artery disease / chronic venous insufficiency	3.238 (0.523–20.046)	0.206
Atrial fibrillation	0.497 (0.148–1.669)	0.258

SD, spondylodiscitis; CS, cervical spine; TS, thoracic spine; LS, lumbar spine; BMI, body mass index; OR, odds ratio; CI, confidence interval. Bold values are significant results (*p* < 0.05) as indicated in the methods.

Other factors such as age, gender, type of infection (SD, ISEE), localization at the spine, MSSA infection, paravertebral psoas abscess, pleural abscess, sepsis, meningism, obesity (BMI > 30 kg/m^2^), immunosuppression, diabetes mellitus, malignancy, dialysis, history of stent or vascular prosthesis, artificial heart valve replacement, peripheral artery disease or chronic venous insufficiency, and atrial fibrillation showed no significant difference in multivariate analysis.

## 4 Discussion

The main findings of this study were that hepatic cirrhosis, septic embolism, and infections with *Streptococcus* spp. and *Enterococcus* spp. were significant independent risk factors for the co-occurrence of IE and PSI. Furthermore, meningism, sepsis and pleural abscesses were found more frequently in PSICIE patients. IE occurred almost exclusively in SD, with MSSA being the most common pathogen and aortic valve IE being the most common type of IE in PSICIE subpopulation. All pathogens were GPB and could be detected via blood cultures up to 95.8%. The modified Duke criteria revealed a sensitivity of 100% and a specificity of 66.7% in the diagnosis of PSICIE patients.

The age and sex distribution, with a predominance of the male sex in both groups in our collective, was similar to that in previous studies (6, 7). Our study is, to the best of our knowledge, the first in which the two main representatives of PSI were assessed separately (SD and ISEE) with respect to IE. We were able to show that IE occurred almost exclusively in SD patients (95.8%).

Similar to the previous study, MSSA was the most common detected causative pathogen in both groups, while *Streptococcus* spp. and *Enterococcus* spp. were observed more frequently in the PSICIE group compared to the PSIWIE group (7, 27). In our study, the PSICIE group was found only in infections with GPB, as in a previous study (27). The aortic valve (50.0%), followed by the mitral valve (20.0%), was the most frequently affected heart valve, as in the previous study (7).

In our study, no pathogen could be detected in only 6.8% of PSI patients, which is substantially better than the existing literature because our collective underwent multiple blood culture tests, open surgery, and additionally CT-guided biopsy of the paravertebral psoas abscess in 53.6% (28, 29). Previous studies identified the pathogens by blood cultures, open surgery, or CT-guided biopsies. Sometimes a combination of two methods was used, but never all three simultaneously, which obviously improved the results.

We observed, as in Viezens et al. study, a significantly higher diagnostic sensitivity for blood cultures in the PSICIE group compared with the PSIWIE group (95.8% vs. 62.4%) (7), which can probably be explained by the vegetation with consequent bacteremia; in contrast, there were no differences between the two groups for intraoperative sampling and CT-guided biopsy. The prompter detection of pathogens via blood cultures in the PSICIE group could perhaps explain why this group was managed more frequently with TAT.

Pleural abscesses were more common in the PSICIE group than in the PSIWIE group, which has not been previously studied in the literature. In contrast, the distribution of paravertebral psoas abscess was similar in both groups. The localization of PSI did not differ between the PSICIE and PSIWIE groups and is consistent with the results in the literature (28, 30).

The PSIECIE and PSIWIE groups were similarly surgically managed by either exclusive abscess evacuation or one- or two-stage surgery. The PSICIE patients spent more time in the hospital but not in the ICU, while the duration of intravenous antibiotic administration and the total duration of antibiotic administration were longer in the PSICIE group than in the PSIWIE group, similar to previous studies (6, 27).

We found more patients with hepatic cirrhosis in the PSICIE than in the PSIWIE group, and multivariate analysis showed that, in addition to septic embolism and pathogen type, hepatic cirrhosis influenced the co-occurrence of IE and PSI. This relevant information has not been reported or included in modified Duke criteria until now. Aagaard et al. observed an increased risk of death from hepatic cirrhosis in patients with spondylodiscitis in his study (31). In nearly one-third of patients with cirrhosis, bacterial infection is present at hospital admission or develops during hospitalization (32), and the mortality due to bacterial infection is four times higher in patients with cirrhosis than in patients without cirrhosis (33).

Sepsis, septic embolism, and meningism occurred more frequently in the PSICIE than in the PSIWIE group in our study, but only septic embolism was an independent factor for the development of IE, which was rightly included in the modified Duke criteria (17).

To date, no multivariate analysis has been performed for the PSICIE subpopulation. Our multivariate analysis highlighted the importance of hepatic cirrhosis, septic embolism, and the presence of *Streptococcus* spp. and *Enterococcus* spp. in PSICIE patients.

Our study showed that the modified Duke criteria have high sensitivity and low specificity for PSICIE subpopulation, which has not been studied before and needs to be modified. Our data showed a significant difference between the PSICIE and PSIWIE group in terms of meningism, sepsis, septic embolism, hepatic cirrhosis, the presence of *Streptococcus* spp. and *Enterococcus* spp. and pleural abscess.

## 5 Limitations and strengths of this study

The monocentric, retrospective nature of our analysis, the long inclusion interval, and the limited number of the PSICIE group (24 patients) might reduce the external validity of our observations. Our study might also be affected by a possible selection bias, e.g., more severe cases due to transfers from surrounding hospitals and the high degree of specialization at our university center. However, our cohort analysis is based on a 20-year treatment period of SD and ISEE in a large university center for neurosurgery, suggesting a high internal validity of our study. Therefore, our observations may be useful to understand the clinical, microbiologic, and radiologic characteristics of the PSICIE subpopulation and the applicability of the modified Duke criteria in PSI patients.

## 6 Conclusions

In the understudied population of patients with PSI and concomitant IE, we identified distinct clinical, radiological, and microbiological phenotypes and we were able to confirm the diagnostic accuracy of the modified Duke criteria in these patients. The pathogen MSSA and the aortic valve as structural organ target seem to play an important role in the pathophysiology of PSICIE patients.

Moreover, we identified specific risk factors for co-occurrence of IE and PSI, including hepatic cirrhosis, septic embolism, and infections with *Streptococcus* spp. and *Enterococcus* spp. Pleural abscess, meningism, sepsis and hepatic cirrhosis might play an essential role in improving the modified Duke criteria in PSI patients and should be investigated in a prospective design.

## Data Availability

The original contributions presented in the study are included in the article Material, further inquiries can be directed to the corresponding author.
